# Do Weight Changes Affect the Association between Smoking Cessation and the Risk of Stroke Subtypes in Korean Males?

**DOI:** 10.3390/ijerph20064712

**Published:** 2023-03-07

**Authors:** Seulji Moon, Yeun Soo Yang, Heejin Kimm, Keum Ji Jung, Ji Young Lee, Sun Ha Jee, Sunmi Lee, So Young Kim, Chung Mo Nam

**Affiliations:** 1 Department of Medicine, College of Medicine, Yonsei University, Seoul 03722, Republic of Korea; 2 Department of Epidemiology and Health Promotion, Institute for Health Promotion, Graduate School of Public Health, Yonsei University, Seoul 03722, Republic of Korea; 3 Department of Public Health, Yonsei University, Seoul 03722, Republic of Korea; 4 Health Insurance Policy Research Institute, National Health Insurance Service, Wonju 26464, Republic of Korea

**Keywords:** smoking cessation, weight gain, stroke

## Abstract

(1) Background: We investigated whether weight changes affect the association between smoking cessation and stroke risk; (2) Methods: Overall, 719,040 males were categorized into eight groups according to smoking status (sustained smokers, non-smokers, long-term quitters (quit > 4 years), and recent quitters (quit < 4 years)) and post-cessation weight change (−5 kg, −5.0 to 0.1 kg, maintainers, 0.1–5.0 kg, and >5.0 kg). The hazard ratios (HR) and 95% confidence intervals (CI) for incident total, ischemic, and hemorrhagic strokes, including subarachnoid and intracerebral hemorrhage, were calculated using Cox proportional hazard models; (3) Results: We detected 38,730 strokes (median follow-up, 25.7 years), including 30,609 ischemic and 9055 hemorrhagic strokes. For recent quitters with a >5.0 kg or 0.1–5.0 kg weight increase, maintainers, or those who lost 0.1–5 kg, the multivariable HR for total stroke was 0.73 (95% CI, 0.67–0.79), 0.78 (95% CI, 0.74–0.82), 0.77 (95% CI, 0.69–0.85), 0.84 (95% CI, 0.77–0.90), and 1.06 (95% CI, 0.92–1.23), respectively, compared with that of sustained smokers; (4) Conclusions: Comparable patterns were obtained for stroke subtypes. Thus, we strongly recommend quitting smoking, as weight gain after quitting smoking does not alter the stroke-related benefits.

## 1. Introduction

Stroke is a major cause of disability and mortality worldwide [1,2,3]. According to the 2017 Global Burden of Diseases, Injuries, and Risk Factors Study, stroke is ranked third among all global causes of death and disability, and is the second-leading cause of death. Additionally, a slow decline in age-standardized stroke incidence has been reported between 1990 and 2017 [3,4]. The lifetime risk of stroke has not decreased since 1990, but has, rather, increased steadily [1]. Moreover, East Asian nations have been experiencing an increase in the aging population, while changes in lifestyle and eating habits, due to rapid industrialization, urbanization, and automation, have led to distinct patterns in stroke risk factor profiles [5]. The main risk factors for stroke include hypertension, active smoking, obesity, poor eating habits, inadequate exercise, and diabetes [6,7,8].

Tobacco smoking is a modifiable risk factor for all stroke subgroups (including hemorrhagic and ischemic strokes) [9,10,11]. Moreover, smoking cessation lowers the risk of stroke [12,13,14]. In contrast, smoking after a stroke increases the risk of stroke recurrence, with the amount of smoking directly influencing the stroke risk [15,16]. Therefore, controlling these risk factors is essential for preventing stroke and lowering the risk of other cardiovascular diseases (CVD).

Smoking cessation can reduce the risk of stroke; however, it is often accompanied by weight changes [17,18]. According to a meta-analysis, 16–17% of individuals who quit smoking lose weight in the first year, whereas approximately 80% gain weight [19]. The average weight increase among these individuals is reportedly 4.10 kg, with a significant 2.61 kg difference between smokers and quitters [17]. Moreover, weight independently influences the incidence of a stroke. There is an increase in stroke risk with a considerable decrease or increase in weight, resulting in a U-shaped relationship between the two variables [20,21,22].

Given that tobacco smoking and weight changes represent distinct risk factors for stroke [23], the current study aimed to determine whether there is a change in the risk of stroke when considering both factors. Previous studies have exclusively focused on weight increases following smoking cessation [24,25], whereas we examined the association of weight reduction with smoking cessation as well. Moreover, unlike myocardial infarction (MI), caused by large-vessel atherosclerotic disease affecting the coronary arteries, identifying stroke risk is challenging because of its complex etiology, resulting in multiple stroke subtypes [6]. Bogiatzi et al. classified stroke subtypes, but only investigated ischemic stroke [26]. Similarly, Grau et al. divided stroke subtype into five categories; however, they did not include hemorrhagic stroke subtypes, such as subarachnoid hemorrhage (SAH) and intracerebral hemorrhage (ICH). Therefore, to explore the effects of each stroke subtype, we analyzed ischemic and hemorrhagic stroke and their subsets (SAH and ICH). Our research question was, “Do weight changes affect the association between smoking cessation and the risk of stroke subtypes in Korean males?” The primary aim of this study was to demonstrate the association between tobacco smoking cessation in the long-term and recent quitting, increased and decreased body weight, and each stroke subtype, including SAH and ICH.

## 2. Materials and Methods

### 2.1. Study Population

The study participants were recruited from the Korean Cancer Prevention Study (KCPS) [27,28]. The KCPS cohort comprised insured individuals who were government employees and the staff of private schools. Participants were enrolled with the Korean Medical Insurance Corporation (currently the National Health Insurance Service (NHIS)) as members of the government employees’ union and private school staff union. Participants who underwent biennial physical examinations at least once between 1992 and 1999 were enrolled in the KCPS cohort [27,28].

**Figure 1:**
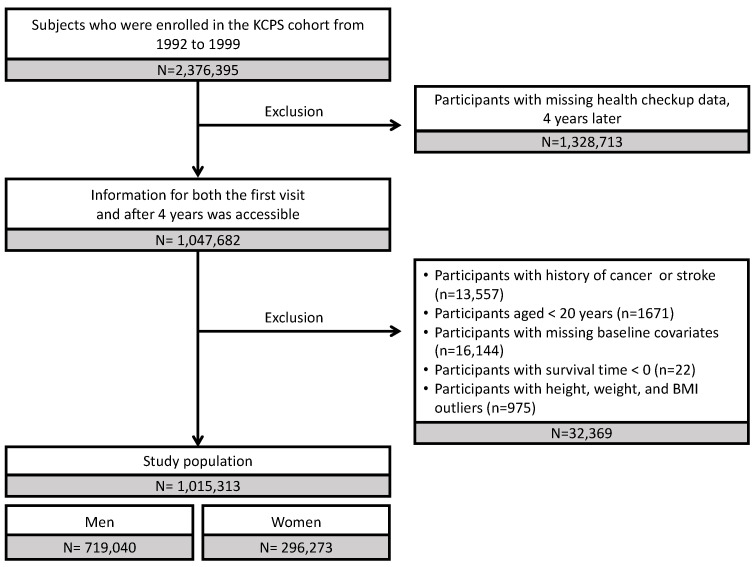
Flow chart of the study population. KCPS, Korean Cancer Prevention Study.

Study participants were selected from 2,376,395 individuals who enrolled in the KCPS between 1992 and 1999. Among these, 1,047,682 individuals with health checkup information available 4 years after their initial visit were included in this study. The exclusion criteria were as follows: history of cancer or stroke (*n* = 13,557), age < 20 years (*n* = 1671), missing baseline data (*n* = 16,144), and survival time < 0 days (*n* = 22). In addition, we disregarded outliers regarding height, weight, and body mass index (BMI; *n* = 975). By examining the scatter plots for height and weight, we confirmed that neither of the variables exhibited a normal distribution. Each variable was log-transformed, and outliers were defined as those with values that deviated from the mean by more than six times the standard deviation. Individuals with an extreme BMI (<16 and >100 kg/m^2^) were excluded (Figure 1). Moreover, females were excluded from the primary analysis, as they comprised approximately 30% of the overall study population, of whom 98% were non-smokers. Finally, the analysis included 719,040 males.

### 2.2. Data Collection and Definitions of Covariates

Participants were required to complete self-reported questionnaires created by the NHIS about their basic lifestyles and undergo standard examinations [29]. They were instructed to respond to questions on smoking history (never, former, or current), daily alcohol consumption (g/day: ethanol), participation in exercise (yes, no), medical history, including diabetes (yes, no), hypertension (yes, no), hyperlipidemia (yes, no), stroke (yes, no); and family medical history, including cancer (yes or no) and stroke (yes or no). Participants were instructed to remove their shoes and wear thin clothes for weight and height measurements. BMI was calculated by dividing the weight (kg) by the square of height (m^2^). BMI was then classified according to the World Health Organization Asia-Pacific classification; individuals were defined as “underweight” (BMI < 18.5 kg/m^2^), “normal” (18.5 ≤ BMI ≤ 22.9 kg/m^2^), “overweight” (23.0 ≤ BMI ≤ 24.9 kg/m^2^), or “obese” (BMI ≥ 25 kg/m^2^) [30]. Blood pressure was measured using standard mercury or an automatic sphygmomanometer in a sitting position [27,31]. Fasting blood specimens were collected and assayed for total cholesterol levels. Baseline blood sample characteristics and patient history, determined by self-reported responses, were used to determine disease history, which was analyzed as a covariate. Hyperlipidemia, diabetes, and hypertension were defined as total cholesterol levels ≥ 200 mg/dL, fasting blood glucose level ≥ 126 mg/dL, and systolic blood pressure (SBP) ≥ 140 mm Hg or diastolic blood pressure (DBP) ≥ 90 mm Hg, respectively.

### 2.3. Definition of Main Outcome and Exposure

The primary outcome of the study included stroke and its subtypes (hemorrhagic, ischemic, or unspecified stroke). Hemorrhagic stroke included SAH and ICH. ICD-10 codes for SAH (I60), ICH (I61), ischemic hemorrhage (I63), and unspecified strokes (I64) have been validated previously [32,33,34,35,36]. Follow-up was conducted from 1992–1999, to 31 December 2019. During follow-up, disease outcome variables were ascertained using the NHIS. Abstractors coded incident disease cases according to the International Statistical Classification of Diseases and Related Health Problems, Tenth Revision (ICD-10).

**Figure 2:**
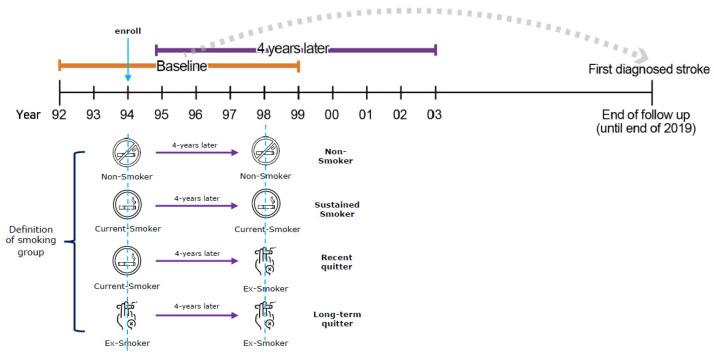
Definition of smoking groups according to smoking status.

Our main focus, smoking status, was defined as follows. Participants enrolled in the study between 1992 and 1999 were defined in terms of smoking status 4 years after enrollment. For instance, by comparing the smoking status 4 years after enrollment, based on the study enrollment period (1995 (for the 1992 study participants) and 1996 (for the 1993 study participants)), data were used to determine the subjects’ smoking status (Figure 2). We evaluated participants according to their self-reported smoking status based on three choices: never smoker, former smoker, or current smoker. Based on self-reported questionnaire responses from the first to third visit after 4 years, smokers were divided into four categories (sustained smokers, non-smokers, long-term quitters, and recent quitters). Sustained smokers and non-smokers were defined as individuals who responded with “current smoker” or “never a smoker” during both visits, respectively. Those who had quit smoking were divided into long-term and recent quitters; the former were defined as those who responded with “former smoker” during both visits, while the latter were defined as those who changed their response from “current smoker” during the first visit to “former smoker” during the third visit. Recent quitters were divided into five groups based on the degree of weight change, by comparing the subjects’ weight between the first and third visits. Individuals with weight changes between −0.1 kg and +0.1 kg were classified as maintainers; between +/−0.1 kg and +/−5.0 kg as mild gainers/losers; greater than +/−5 kg as severe gainers/losers.

### 2.4. Statistical Analyses

This study is an observational study with a long-term retrospective cohort. It was conducted following the Strengthening the Reporting of Observational Studies in Epidemiology guidelines. We evaluated the effect of smoking cessation and weight change on stroke risk using sustained smokers as the reference group. Person-years were estimated from the beginning of the study to the end, considering the date of death, loss to follow-up, or end of the study, depending on which occurred first. Estimated hazard ratios (HR) and 95% confidence intervals (CIs) for smoking status and stroke incidence were calculated using Cox proportional hazards regression models. The Cox proportional hazards hypothesis was evaluated using log cumulative hazard graphs and time-dependent coefficients in Cox models. HRs were calculated from two different models: a basic one with adjustment for age and BMI (Model 1), and a multivariate one (Model 2) with adjustment for age, BMI, exercise, alcohol consumption, medical history (diabetes, hyperlipidemia, and hypertension), and family medical history (cancer and stroke). All outcomes were for males, and the HRs were reported for both models. Statistical calculations and analyses were performed using SAS version 9.4 (SAS Institute Inc., Cary, NC, USA), and data were visualized using R 4.0.5 (R Core Team, Vienna, Austria). *p*-values < 0.05 were considered statistically significant.

## 3. Results

### 3.1. Baseline Characteristics

Table 1 shows the baseline characteristics of the study participants according to smoking and weight gain/loss statuses. Among the 719,040 males enrolled in the study, 60.2% were sustained smokers, followed by non-smokers (23.3%), and long-term (8.3%) and recent quitters (8.2%). The most prevalent age range within each smoking group was 30–39 years. In the long-term quitter group, the highest proportions of individuals, following the 30–39 year-old group, were observed in 40–49 and 50–59 year-old age groups, accounting for approximately 50.0%. This proportion was higher than those of the corresponding ages in other groups. Sustained smokers comprised 48.0% of overweight or obese populations, whereas long-term quitters comprised 56.5% of obese populations. These results showed long-term quitters were more likely to be overweight than sustained smokers. However, the mean weight change between the two groups at the first visit and after 4 years was not significantly different. Among recent quitters, mild gainers (0.1–5 kg increase) accounted for 41.7%, followed by severe gainers (27.0%) and mild losers (17.0%). Age proportions, according to the weight change of recent quitters, were similar; however, the ratio of those aged 20–29 years in the severe gainer group differed by at least 10% from their corresponding age ratios in the other four groups. Severe losers, including those who lost >5 kg, experienced an average weight change of 7.3 kg; this group comprised the highest percentage of obese individuals. Additionally, this group showed high mean values for all blood parameters, including total cholesterol, SBP, DBP, and fasting blood glucose, as well as having high rates of diabetes, hypertension, and hyperlipidemia in their medical histories.

### 3.2. Association between Smoking Cessation and Stroke Outcomes in Korean Males

First, the risk of stroke according to the smoking cessation period was analyzed. During follow-up, we identified 38,730 stroke events of any type, 30,609 ischemic strokes, 9055 (SAH: 2593 and ICH: 6727) hemorrhagic strokes, and 1390 stroke events of unspecified type. The incidence rates of total stroke were higher among sustained smokers (221 per 100,000 person-years) and long-term quitters (229 per 100,000 person-years) than among non-smokers (176 per 100,000 person-years) and recent quitters (204 per 100,000 person-years). Ischemic stroke had the highest incidence rate, followed by hemorrhagic stroke, subarachnoid hemorrhage, intracerebral hemorrhage, and unspecified stroke (Table 2).

The risk of all stroke types was lower among non-smokers, long-term quitters, and recent quitters than among sustained smokers. After multivariable adjustment, the risk of total (HR 0.62, 95% CI 0.60–0.64), ischemic (HR 0.59, 95% CI 0.57–0.61), hemorrhagic (HR 0.74, 95% CI 0.69–0.80), and unspecified stroke (HR 0.81, 95% CI 0.68–0.97) was significantly lower for long-term quitters. Similarly, after multivariate adjustment for recent quitters, the risk of total (HR 0.79, 95% CI 0.76–0.82), ischemic (HR 0.78, 95% CI 0.74–0.81), and hemorrhagic (HR 0.80, 95% CI 0.74–0.69–0.87) strokes were significantly reduced. Among recent quitters, the risk of all other types of stroke was significantly lower in Model 1 (adjusted for age and BMI) and Model 2 (adjusted for all covariates), except for undefined stroke (Table 3). These protective results were also observed among recent quitters; however, recent quitters exhibited fewer positive health effects than long-term quitters.

### 3.3. Association between Weight Changes in Recent Quitters and Stroke Outcomes in Korean Males

Table 4 shows stroke incidence rates in the subgroups of recent quitters based on weight changes in Korean males. Regarding total stroke, severe losers had the highest total number of stroke incidences (377 per 100,000 person-years), and severe gainers had the lowest (138 per 100,000 person-years). Regarding stroke subtypes, severe losers had the highest ischemic stroke incidence rate (309 per 100,000 person-years), and severe losers had the lowest ischemic stroke incidence rate (107 per 100,000 person-years). The incidence rate of hemorrhagic and ischemic stroke was similar in all groups, with severe losers having the highest hemorrhagic stroke incidence rate (68 per 100,000 person-years) and severe gainers having the lowest incidence rate (31 per 100,000 person-years). The table also shows that SAH was less common than ICH, with severe losers having the highest SAH incidence rate (30 per 100,000 person-years) and severe gainers having the lowest incidence rate (10 per 100,000 person-years). Finally, the table shows the incidence of unspecified stroke, with severe losers having the highest rate (18 per 100,000 person-years) and severe gainers having the lowest rate (6 per 100,000 person-years) (Table 4).

**Figure 3:**
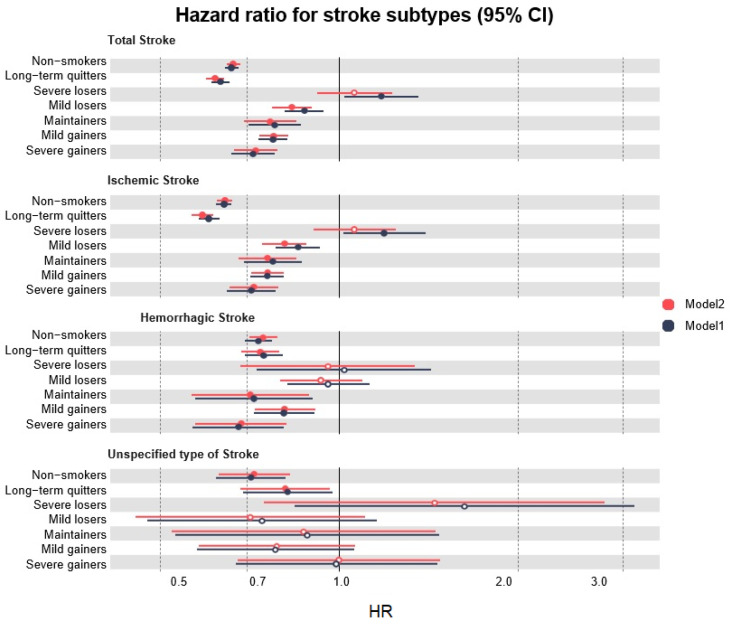
Multivariate models showing the effect of smoking cessation and weight changes in recent quitters on stroke subtypes. 95% CI, 95% confidence interval; HR, Hazard Ratio.

We assessed the risk of stroke in the recent quitters’ subgroup, based on weight changes in Korean males. We investigated the results of Model 1 after adjusting for age and BMI, and observed differences among the weight change findings in the recent quitters’ subgroup. The detailed HR values and 95% CIs are shown in Supplemental Table S1, and the overall results are represented graphically (Figure 3). The weight change outcomes in all subgroups were statistically significant for total stroke incidence. An overall decrease in the risk of total stroke was observed in the weight change groups when severe losers were excluded (Figure 3). Among these subgroups, severe gainers had the lowest risk (HR 0.72, 95% CI 0.66–0.78), followed by mild gainers (HR 0.78, 95% CI 0.72–0.82), maintainers (HR 0.78, 95% CI 0.71–0.87), and mild losers (HR 0.88, 95% CI 0.81–0.94). After multivariate adjustment, a significant reduction in the risk of total stroke was detected among severe gainers (HR 0.73, 95% CI 0.67–0.79), followed by mild gainers (HR 0.78, 95% CI 0.74–0.82), maintainers (HR 0.77, 95% CI 0.69–0.85), and mild losers (HR 0.84, 95% CI 0.77–0.90) (Supplemental Table S1). These results were similar to the trends of ischemic and hemorrhagic strokes; severe gainers experienced the most protective association among all weight change subgroups (Figure 3).

In addition, we examined the results, focusing on weight loss. Severe weight loss of >5 kg was associated with a higher risk of total (HR 1.18, 95% CI 1.02–1.36) and ischemic stroke (HR 1.19, 95% CI 1.02–1.40), after adjusting for age and BMI. In addition to hemorrhagic stroke, its subtypes (SAH and ICH), and unspecified stroke, severe weight loss was associated with an increased risk of health outcomes, but it was not significant. Among unspecified strokes, no significant results were observed according to the weight change subgroups. Therefore, we conducted a sensitivity analysis by omitting patients who were obese at baseline. The findings of the sensitivity analysis supported the trends of the main study (Supplemental Tables S2–S4).

## 4. Discussion

Using a large prospective cohort study dataset for Korean males, we confirmed a reduced risk of stroke and its subtypes in individuals who quit tobacco smoking when compared with sustained smokers. This study confirms that weight gain after tobacco smoking cessation alters the health benefits of smoking cessation, in terms of the incidence of certain types of strokes. This stroke-related health benefit of quitting tobacco smoking persisted despite weight gain after smoking cessation. These findings were corroborated by a meta-analysis of similar cohort studies, which reported that weight gain in quitters did not reduce the protective association against stroke [13].

There were two significant disparities between our study and previous studies [24,25]. In previous studies, participants were divided into weight gain and no weight gain groups, and the total incidence of stroke was reported. However, our study classified weight loss into severe and mild weight loss groups, while also reporting the stroke subtypes. A follow-up study of 69,910 Japanese individuals with an average follow-up period of 14.8 years reported that the risk of stroke after weight gain following smoking cessation was 0.75 (95% CI 0.52–1.09), which was not significant; however, it is comparable to our study [24]. The results of this study are comparable to ours, because it focused on a particular subset of smoking status and included a large sample size, long follow-up duration, and stroke outcome analysis. However, no direct weight loss analysis was conducted, and the results of no weight gain, including that of the weight maintenance group, were insignificant (0.81; 95% CI 0.59–1.10).

Our results can also be compared with those of a Korean population cohort study that assessed total stroke, which was further categorized into ischemic and hemorrhagic stroke groups, as the outcome, and applied changes in BMI, which may be related to weight change, as the main exposure [14]. Previous studies have shown similar trends in outcomes for ischemic and hemorrhagic strokes after weight gain due to smoking cessation; however, none of the subtypes, including a total stroke risk of 0.75 (95% CI 0.57–1.00), was statistically significant [14]. Thus, the relationship could not be defined.

In this study, smoking cessation reduced the risk of stroke, and this protective effect was maintained regardless of weight maintenance or increase after smoking cessation. This might explain the etiologic mechanism by which CVD exerts its protective effect, despite the deteriorating metabolic status due to weight gain after smoking cessation. Smoking, particularly nicotine, has been linked to higher levels of triglycerides, low-density lipoproteins (LDL), high-density lipoproteins (HDL), and postprandial dyslipidemia, with higher levels of insulin resistance and atherosclerosis [37,38]. This is primarily caused by tobacco smoking-induced increases in the amount of catecholamines in the blood, resulting in an increased lipolysis, free fatty acid release into the blood, and lipoprotein formation, particularly LDL, which promotes atherosclerosis [39]. Consequently, smokers have higher plasma levels of LDL and triglycerides, lower levels of HDL, and higher levels of oxidized LDL, which are preferentially taken up by macrophages, and are essential for the formation of the foam cells observed in atherosclerotic lesions [38,40]. Therefore, the benefits of quitting smoking on lipid profiles, such as LDL, triglycerides, HDL, and oxidized LDL levels, are more noticeable with an increased duration of smoking cessation [41,42]. However, no significant changes were reported in the oxidized LDL levels in smokers who gained weight, because oxidative stress increases with weight gain, contributing to the attenuation of reduced oxidized LDL levels [43]. Therefore, we inferred that oxidized LDL levels do not change, even with weight gain. This validates the finding that no modified CVD risks are associated with smoking cessation, even if weight increases.

In this study, when smokers lost >5 kg, the HR for stroke was 1.18 (95% CI 1.02–1.36) in the model adjusted for age and BMI for total stroke, and was 1.06 (95% CI 0.92–1.23) in the multivariate model. Weight reduction is desirable to maintain a healthy form [44]. However, several studies have reported that weight loss increases the chance of developing various health outcomes, such as CVD, cancer, and death [45,46]. A large-scale prospective cohort study of Koreans revealed an increased risk ratio for death when the BMI is <18.5 [28]. A meta-analysis confirmed that weight loss increased the risk of CVD and death [47]. This pattern of increased risk might be because of bias caused by weight loss resulting from wasting illnesses, such as cardiovascular illness, cancer, renal disease, or chronic obstructive pulmonary disease [48]. Weight loss decreases body fat and causes the body to lose considerable nutrients and water [49]. Additionally, it causes diuresis, resulting in a major loss of magnesium, calcium, and phosphorus [50]. Thus, severe weight loss might increase health risks because of nutritional deficiencies [49]. Our study showed that despite smoking cessation, the risk of total and ischemic strokes increased considerably in the severe losers’ group, but only in the model adjusted for age and BMI. However, the difference was not significant when we controlled for factors such as physical activity, alcohol use, medical history (diabetes, hyperlipidemia, and hypertension), and family medical history (cancer and stroke). Hence, weight loss due to these correcting variables or other conditions might increase the risk of stroke during smoking cessation, in people with a weight loss of >5 kg. Considering that unintentional or intentional excessive weight loss can influence mortality, we categorized participants into five subgroups based on their weight change status, using a 5 kg change as the standard, in accordance with a previous study [16].

However, the current study has certain limitations. First, we only included males, as the small number of females that met the inclusion criteria made it difficult to perform a complete analysis based on stroke subtype and weight changes. Second, recent quitters were defined as those who quit smoking within the 4 years we examined, whereas long-term quitters were defined as those who reported past smoking in both surveys. According to the two survey periods, we assumed that the smoking cessation time for recent quitters was a maximum of 4 years, whereas it was at least 4 years for long-term quitters. However, since these were operationally defined by the follow-up interval of every 2 years, they cannot be quantified as continuous variables; hence, they are considered limitations, as they were self-reported and subject to bias. Furthermore, a distinct risk difference was anticipated, due to the gap of at least 1 year between the two groups regarding smoking cessation. Third, this study did not consider the details of smoking behavior, such as the number of cigarettes smoked, the smoking duration, or types of tobacco products. The use of novel tobacco products, such as e-cigarettes and heated tobacco, has increased in recent years; however, it is also important to emphasize the benefits of tobacco smoking cessation. This is because studies have shown that e-cigarettes lower the risk of CVD by 30–40% more than cigarette smoking [51], implying that cigarette smoking is more harmful than e-cigarettes. Fourth, we did not consider the various factors that may affect weight change. Participants with a history of cancer and stroke were excluded, but we could not consider additional underlying diseases because of limited information. Finally, since the KCPS data used in the analysis were obtained from a cohort of individuals who underwent health examinations every 2 years; the study population comprised individuals who led relatively healthy lives and those who were interested in health maintenance. Hence, the issue of selection bias could not be completely addressed [52]. Nevertheless, including a sizable sample size, wide age range, and long-term follow-up data for Asians, particularly Koreans, is noteworthy.

The advantage of this study is that it examined both weight gain and loss while considering potential weight changes in individuals who had quit smoking. In addition, it is a large-scale cohort study reporting long-term prospective risk findings of total stroke, ischemic stroke, hemorrhagic stroke, and the specific subtypes ICH and SAH. Further, this study characterized short- and long-term quitter groups in accordance with previous studies [53]. This classification is necessary, as there are differences in the health outcomes of short- and long-term quitters. The risk of stroke decreases after 2 to 4 years of quitting and is similar to that of non-smokers after 5 years [8,54]. Thus, we must conduct an analysis that considers the influence of the duration after smoking cessation.

According to the findings of this study, maintaining or gaining weight after smoking cessation appears to have a protective association and does not increase the risk of total stroke, hemorrhagic stroke, SAH, or ICH, except for unspecified stroke, which includes special circumstances, such as the absence of neuroimaging, non-referral by the primary care physician, death before reaching an imaging device, or refusal of additional treatment by the patient [55]. Because of worries about weight gain, half of the female smokers and a quarter of male smokers do not attempt to quit smoking [56]. Our research hypothesis was tested to determine whether weight changes affect the association between smoking cessation and the risk of stroke subtypes in Korean males. However, the results indicated that weight change did not alter the protective effect of smoking cessation on stroke. That is, the reluctance to quit tobacco use, owing to fear of weight gain, will not be beneficial for preventing stroke, and this evidence provides a basis to strongly promote smoking cessation. Finally, it is expected that this study will provide essential information for the population needing a thorough long-term follow-up prognosis for stroke, following smoking cessation and weight change.

## 5. Conclusions

Weight gain following smoking cessation does not alter the health advantages of smoking cessation for stroke and its subtypes. Thus, the reluctance to quit smoking owing to fear of weight gain will not be beneficial for preventing stroke.

## Figures and Tables

**Table 1:** Baseline characteristics of the study population.

	Sustained Smokers (*N* = 433,099)	Non- Smokers (*N* = 167,716)	Long-Term Quitters (*N* = 59,311)	Recent Quitters (*N* = 58,914)				
				Severe Losers (*N* = 2063)	Mild Losers (*N* = 10,000)	Maintainers (*N* = 6362)	Mild Gainers (*N* = 24,577)	Severe Gainer (*N* = 15,912)
Age, years								
20–29	111,957 (25.8)	40,689 (24.3)	5901 (9.9)	327 (15.8)	1594 (15.9)	950 (14.9)	4595 (18.7)	5201 (32.7)
30–39	163,248 (37.7)	59,670 (35.6)	19,012 (32.1)	667 (32.3)	3403 (34.0)	2302 (36.2)	9567 (38.9)	5979 (37.6)
40–49	97,175 (22.4)	37,196 (22.2)	18,619 (31.4)	525 (25.5)	2669 (26.7)	1714 (26.9)	6390 (26.0)	3124 (19.6)
50–59	51,340 (11.9)	25,306 (15.1)	12,116 (20.4)	423 (20.5)	1886 (18.9)	1181 (18.6)	3336 (13.6)	1344 (8.4)
≥60	9379 (2.2)	4855 (2.9)	3663 (6.2)	121 (5.9)	448 (4.5)	215 (3.4)	689 (2.8)	264 (1.7)
BMI, kg/m^2^								
Underweight	8225 (1.9)	3037 (1.8)	840 (1.4)	18 (0.9)	166 (1.7)	102 (1.6)	424 (1.7)	327 (2.1)
Normal	217,128 (50.1)	78,654 (46.9)	24,988 (42.1)	630 (30.5)	4158 (41.6)	2889 (45.3)	11,683 (47.6)	8991 (56.5)
Overweight	115,970 (26.8)	47,873 (28.5)	18,187 (30.7)	495 (24.0)	2783 (27.8)	1785 (28.1)	7334 (29.8)	4120 (25.9)
Obese	91,776 (21.2)	38,152 (22.8)	15,296 (25.8)	920 (44.6)	2893 (28.9)	1586 (25.0)	5136 (20.9)	2474 (15.5)
Weight change, kg	1.2 ± 3.8	1.3 ± 3.7	0.8 ± 3.5	–7.3 ± 4.8	–2.0 ± 1.0	0.0 ± 0.0	2.5 ± 1.1	7.2 ± 2.7
Exercise (yes)	135,077 (31.2)	65,901 (39.3)	22,973 (38.7)	681 (33.0)	3402 (34.0)	2178 (34.2)	8468 (34.5)	5733 (36.0)
Alcohol consumption (yes)	356,341 (82.3)	103,818 (61.9)	44,602 (75.2)	1693 (82.1)	8320 (83.2)	5286 (83.1)	20,289 (82.6)	13,083 (82.2)
Biochemical measurements								
TC, mg/dL	178.9 ± 52.4	178.2 ± 50.7	187.8 ± 46.3	189.5 ± 52.1	184.7 ± 50.5	184.0 ± 48.5	181.9 ± 48.6	176.3 ± 49.4
SBP, mmHg	121.7 ± 13.7	122.9 ± 14.3	124.2 ± 15.1	125.7 ± 17.0	123.4 ± 15.3	122.6 ± 14.5	121.7 ± 13.8	120.7 ± 13.2
DBP, mmHg	79.0 ± 10.1	79.9 ± 10.5	80.9 ± 10.7	81.6 ± 11.7	80.3 ± 10.9	79.8 ± 10.5	79.1 ± 10.3	78.1 ± 9.9
FBS, mg/dL	86.1 ± 26.9	86.8 ± 26.4	90.1 ± 25.1	97.9 ± 40.2	90.7 ± 30.7	88.4 ± 26.0	86.5 ± 24.4	84.8 ± 24.1
History (yes)								
Diabetes	18,255 (4.2)	6953 (4.2)	3678 (6.2)	387 (18.7)	961 (9.6)	372 (5.9)	1015 (4.1)	426 (2.7)
Hypertension	121,994 (28.2)	52,379 (31.2)	21,481 (36.2)	805 (39.0)	3352 (33.5)	2013 (31.6)	7066 (28.8)	3958 (24.9)
Hyperlipidemia	137,567 (31.8)	51,324 (30.6)	22,281 (37.6)	804 (39.0)	3612 (36.1)	2189 (34.4)	8070 (32.8)	4475 (28.1)
Family health history (yes)								
Cancer	76,170 (17.6)	29,119 (17.4)	12,858 (21.7)	381 (18.5)	2112 (21.1)	1348 (21.2)	5077 (20.7)	2963 (18.6)
Stroke	65,848 (15.2)	25,886 (15.4)	11,924 (20.1)	378 (18.3)	1808 (18.1)	1164 (18.3)	4317 (17.6)	2287 (14.4)

Continuous variables are presented as mean ± standard deviation (SD); categorical values are presented as *n* (%). Percentages may not equal 100 because of rounding. BMI, body mass index; TC, total cholesterol; SBP, systolic blood pressure; DBP, diastolic blood pressure; FBS, fasting blood glucose.

**Table 2:** Incidence rates of stroke and its subtypes (per 100,000 person-years) according to smoking cessation in Korean males.

	Sustained Smokers (*N* = 433,099)	Non- Smokers (*N* = 167,716)	Long-Term Quitters (*N* = 59,311)	Recent Quitters (*N* = 58,914)
Total stroke (*N* = 38,730)				
Number of events	24,449	7688	3516	3077
Person-years	11,077,402	4,357,065	1,532,314	1,509,428
Incidence rates per 100,000 PY	221	176	229	204
Ischemic stroke (*N* = 30,609)				
Number of events	19,347	6048	2778	2436
Person-years	11,115,801	4,370,176	1,538,171	1,514,985
Incidence rates per 100,000 PY	174	138	181	161
Hemorrhagic stroke (*N* = 9055)				
Number of events	5697	1839	831	688
Person-years	11,219,801	4,402,400	1,551,886	1,528,424
Incidence rates per 100,000 PY	51	42	54	45
SAH (*N* = 2593)				
Number of events	1813	386	201	193
Person-years	11,245,095	4,412,865	1,556,477	1,532,183
Incidence rates per 100,000 PY	16	9	13	13
ICH (*N* = 6727)				
Number of events	4076	1487	652	512
Person-years	11,233,742	4,405,377	1,553,344	1,530,057
Incidence rates per 100,000 PY	36	34	42	33
Unspecified type of stroke (*N* = 1390)				
Number of events	830	286	160	114
Person-years	11,255,086	4,414,175	1,557,226	1,533,050
Incidence rates per 100,000 PY	7	6	10	7

PY, person year; SAH, subarachnoid hemorrhage; ICH, Intracerebral hemorrhage.

**Table 3:** HR and 95% CI for stroke and its subtypes, according to smoking cessation in Korean males.

	Sustained Smokers † (*N* = 433,099)	Non- Smokers (*N* = 167,716)	Long-Term Quitters (*N* = 59,311)	Recent Quitters (*N* = 58,914)
Total stroke (*N* = 38,730)				
Model 1 HR (95% CI)	1	0.66 (0.64–0.68)	0.63 (0.61–0.66)	0.80 (0.77–0.83)
Model 2 HR (95% CI)	1	0.67 (0.65–0.68)	0.62 (0.60–0.64)	0.79 (0.76–0.82)
Ischemic stroke (*N* = 30,609)				
Model 1 HR (95% CI)	1	0.64 (0.62–0.66)	0.60 (0.58–0.63)	0.79 (0.76–0.82)
Model 2 HR (95% CI)	1	0.64 (0.62–0.66)	0.59 (0.57–0.61)	0.78 (0.74–0.81)
Hemorrhagic stroke (*N* = 9055)				
Model 1 HR (95% CI)	1	0.73 (0.70–0.77)	0.75 (0.69–0.80)	0.81 (0.75–0.87)
Model 2 HR (95% CI)	1	0.75 (0.71–0.79)	0.74 (0.69–0.79)	0.80 (0.74–0.87)
SAH (*N* = 2593)				
Model 1 HR (95% CI)	1	0.51 (0.50–0.57)	0.66 (0.57–0.76)	0.74 (0.64–0.86)
Model 2 HR (95% CI)	1	0.51 (0.46–0.57)	0.65 (0.57–0.76)	0.74 (0.64–0.86)
ICH (*N* = 6727)				
Model 1 HR (95% CI)	1	0.81 (0.76–0.86)	0.76 (0.71–0.84)	0.83 (0.75–0.90)
Model 2 HR (95% CI)	1	0.83 (0.78–0.88)	0.76 (0.70–0.83)	0.82 (0.75–0.90)
Unspecified type of stroke (*N* = 1390)				
Model 1 HR (95% CI)	1	0.71 (0.62–0.82)	0.82 (0.69–0.98)	0.87 (0.71–1.05)
Model 2 HR (95% CI)	1	0.72 (0.63–0.83)	0.81 (0.69–0.97)	0.85 (0.70–1.04)

SAH, subarachnoid hemorrhage; ICH, Intracerebral hemorrhage. Model 1: adjusted for age, body mass index; Model 2: further adjusted for exercise, alcohol consumption, medical history (diabetes, hyperlipidemia, hypertension), and family medical history (cancer, stroke); † This group served as the reference group.

**Table 4:** Incidence rates of stroke and its subtypes, according to smoking cessation and weight change in Korean males.

	Sustained Smokers (*N* = 433,099)	Non- Smokers (*N* = 167,716)	Long-Term Quitters (*N* = 59,311)	Recent Quitters (*N* = 58,914)				
				Severe Losers (*N* = 2063)	Mild Losers (*N* = 10,000)	Maintainers (*N* = 6362)	Mild Gainers (*N* = 24,577)	Severe Gainers (*N* = 15,912)
Total number of stroke incidences (*N* = 38,730)								
Number of events	24,449	7688	3516	185	674	381	1273	564
Person-years	11,077,402	4,357,065	1,532,314	49,135	251,738	164,062	635,592	408,902
Incidence rate per 100,000 PY	221	176	229	377	268	232	200	138
Ischemic stroke (*N* = 30,609)								
Number of events	19,347	6048	2778	153	537	307	1000	439
Person-years	11,115,801	4,370,176	1,538,171	49,446	252,882	164,649	637,960	410,048
Incidence rate per 100,000 PY	174	138	181	309	212	186	157	107
Hemorrhagic stroke (*N* = 9055)								
Number of events	5697	1839	831	34	157	75	294	128
Person-years	11,219,801	4,402,400	1,551,886	50,293	255,871	166,409	643,379	412,472
Incidence rate per 100,000 PY	51	42	54	68	61	45	46	31
SAH (*N* = 2593)								
Number of events	1813	386	201	15	39	13	84	42
Person-years	11,245,095	4,412,865	1,556,477	50,403	256,663	166,839	645,119	413,158
Incidence rate per 100,000 PY	16	9	13	30	15	8	13	10
ICH (*N* = 6727)								
Number of events	4076	1487	652	19	123	62	217	91
Person-years	11,233,742	4,405,377	1,553,344	50,493	256,234	166,513	643,987	412,831
Incidence rate per 100,000 PY	36	34	42	38	48	37	34	22
Unspecified type of stroke (*N* = 1390)								
Number of events	830	286	160	9	20	15	44	26
Person-years	11,255,086	4,414,175	1,557,226	50,546	256,913	166,839	645,447	413,305
Incidence rate per 100,000 PY	7	6	10	18	8	9	7	6

PY, person year; SAH, subarachnoid hemorrhage; ICH, Intracerebral hemorrhage.

## Data Availability

Owing to the nature of this study, participants did not agree to share their data publicly; hence, the supporting data are not available.

## Supplementary Materials

The following supporting information can be downloaded at: https://www.mdpi.com/article/10.3390/ijerph20064712/s1, Table S1: Hazard ratio and 95% CI for stroke and its subtypes according to smoking cessation and weight change in Korean males; Table S2: Baseline characteristics of the study population excluding those with obesity; Table S3: Incidence rates of stroke and its subtypes according to smoking cessation and weight change in the study population excluding those with obesity; Table S4: Hazard ratio and 95% confidence interval for stroke and its subtypes according to smoking cessation and weight change in the study population excluding those with obesity.
