# Supplementary material for: Do Weight Changes Affect the Association between Smoking Cessation and the Risk of Stroke Subtypes in Korean Males?
Source: Int J Environ Res Public Health. 2023 Mar 7;20(6):4712. doi: 10.3390/ijerph20064712 (PMC10048944; doi:10.3390/ijerph20064712)
Supplement: Supplementary file 1 [file ijerph-20-04712-s001.zip › ijerph-2221020-supplementary.pdf]

Table S1. Hazard ratio and 95% CI for stroke and its subtypes according to smoking cessation and weight change in Korean males.

	Sustained Smokers † (*N* = 433,099)	Non-Smokers (*N* = 167,716)	Long-Term Quitters (*N* = 59,311)	Recent Quitters (*N* = 58,914)				
				Severe Losers (*N* = 2063)	Mild Losers (*N* = 10,000)	Maintainers (*N* = 6362)	Mild Gainers (*N* = 24,577)	Severe Gainers (*N* = 15,912)
**Total number of stroke incidences (*N* = 38,730)**								
Model 1 HR (95% CI)	1	0.66 (0.64–0.68)	0.63 (0.61–0.66)	1.18 (1.02–1.36)	0.88 (0.81–0.94)	0.78 (0.71–0.87)	0.78 (0.72–0.82)	0.72 (0.66–0.78)
Model 2 HR (95% CI)	1	0.67 (0.65–0.68)	0.62 (0.60–0.64)	1.06 (0.92–1.23)	0.84 (0.77–0.90)	0.77 (0.69–0.85)	0.78 (0.74–0.82)	0.73 (0.67–0.79)
**Ischemic stroke (*N* = 30,609)**								
Model 1 HR (95% CI)	1	0.64 (0.62–0.66)	0.61 (0.58–0.63)	1.19 (1.02–1.40)	0.85 (0.78–0.93)	0.78 (0.69–0.87)	0.76 (0.71–0.81)	0.71 (0.65–0.78)
Model 2 HR (95% CI)	1	0.64 (0.62–0.66)	0.59 (0.57–0.61)	1.06 (0.91–1.25)	0.81 (0.74–0.88)	0.76 (0.68–0.85)	0.76 (0.71–0.81)	0.72 (0.66–0.79)
**Hemorrhagic stroke (*N* = 9055)**								
Model 1 HR (95% CI)	1	0.73 (0.70–0.77)	0.75 (0.70–0.81)	1.02 (0.73–1.43)	0.96 (0.82–1.13)	0.72 (0.57–0.90)	0.81 (0.72–0.91)	0.68 (0.57–0.81)
Model 2 HR (95% CI)	1	0.75 (0.71–0.79)	0.74 (0.69–0.80)	0.96 (0.68–1.34)	0.93 (0.80–1.09)	0.71 (0.57–0.89)	0.81 (0.72–0.91)	0.69 (0.58–0.82)
**SAH (*N* = 2593)**								
Model 1 HR (95% CI)	1	0.51 (0.46–0.57)	0.66 (0.57–0.76)	1.61 (0.97–2.68)	0.83 (0.60–1.14)	0.43 (0.25–0.74)	0.76 (0.61–0.95)	0.67 (0.49–0.90)
Model 2 HR (95% CI)	1	0.51 (0.46–0.57)	0.66 (0.57–0.76)	1.62 (0.97–2.69)	0.83 (0.61–1.14)	0.43 (0.25–0.74)	0.76 (0.61–0.95)	0.67 (0.49–0.91)
**ICH (*N* = 6727)**								
Model 1 HR (95% CI)	1	0.81 (0.76–0.86)	0.78 (0.71–0.84)	0.76 (0.48–1.19)	1.01 (0.84–1.21)	0.81 (0.72–0.94)	0.82 (0.72–0.94)	0.69 (0.56–0.84)
Model 2 HR (95% CI)	1	0.83 (0.78–0.88)	0.76 (0.70–0.83)	0.70 (0.45–1.10)	0.97 (0.81–1.17)	0.79 (0.61–1.02)	0.82 (0.72–0.94)	0.69 (0.56–0.85)
**Unspecified type of stroke (*N* = 1390)**								
Model 1 HR (95% CI)	1	0.71 (0.62–0.82)	0.82 (0.69–0.98)	1.63 (0.84–3.14)	0.74 (0.48–1.16)	0.89 (0.53–1.48)	0.78 (0.58–1.06)	0.99 (0.67–1.46)
Model 2 HR (95% CI)	1	0.72 (0.63–0.83)	0.81 (0.68–0.97)	1.45 (0.75–2.80)	0.71 (0.46–1.11)	0.87 (0.52–1.46)	0.79 (0.58–1.07)	1.00 (0.68–1.48)

SAH, subarachnoid hemorrhage; ICH, Intracerebral hemorrhage. Model 1: adjusted for age and body mass index. Model 2: further adjusted for exercise, alcoholic drinks, medical history (diabetes, hyperlipidemia, hypertension), and family medical history (cancer, stroke). † This group served as the reference group.

Table S2. Baseline characteristics of the study population excluding those with obesity.

	Sustained Smokers (*N* = 341,323)	Non-Smokers (*N* = 129,564)	Long-Term Quitters (*N* = 44,015)	Recent Quitters (*N* = 45,905)				
				Severe Losers (*N* = 1143)	Mild Losers (*N* = 7107)	Maintainers (*N* = 819)	Mild Gainers (*N* = 3967)	Severe Gainer (*N* = 4536)
**Age, years**								
20–29	94,324 (27.6)	35,241 (27.2)	5076 (11.5)	208 (18.2)	1298 (18.3)	819(17.2)	3967 (20.4)	4536 (33.8)
30–39	125,719 (36.8)	46,371 (35.8)	14,706 (33.4)	317 (27.7)	2356 (33.2)	2356 (33.2)	7471 (38.4)	5018 (37.3)
40–49	73,060 (21.4)	26,680 (20.1)	12,955 (29.4)	279 (24.4)	1755 (25.0)	1755 (24.7)	1181 (24.7)	2554 (19.0)
50–59	40,188 (11.8)	17,697 (13.7)	8426 (19.1)	252 (22.1)	1344 (18.9)	1344 (18.9)	860 (18.0)	1095 (8.2)
≥ 60	8032 (2.3)	3575 (2.8)	2852 (6.5)	87 (7.6)	354 (5.0)	354 (5.0)	173 (3.6)	235 (1.7)
**BMI, kg/m^2**								
Underweight	8225 (2.4)	3037 (2.3)	840 (1.9)	18 (1.6)	166 (2.3)	102 (2.1)	424 (2.2)	327 (2.4)
Normal	217,128 (63.6)	78,654 (60.7)	24,988 (56.8)	630 (55.1)	4158 (58.5)	2889 (60.5)	11,683 (60.1)	8991 (66.9)
Overweight	115,970 (34.0)	47,873 (37.0)	18,187 (41.3)	495 (43.4)	2783 (39.2)	1785 (37.4)	7334 (37.7)	4120 (30.7)
Weight change, kg	1.2 ± 3.8	1.3 ± 3.7	0.8 ± 3.5	−7.3 ± 4.8	−2.0 ± 1.0	0.0 ± 0.0	2.5 ± 1.1	7.2 ± 2.7
Exercise (yes)	105,320 (30.9)	50,672 (39.1)	16,739 (38.0)	371 (32.5)	2384 (33.5)	1618 (33.9)	6546 (33.7)	4751 (35.4)
Alcohol consumption (yes)	279,199 (81.8)	78,322 (60.5)	32,701 (74.3)	927 (81.1)	5849 (82.3)	3940 (82.5)	15,965 (82.1)	10,974 (81.7)
**Biochemical measurements**								
TC, mg/dL	175.3 ± 51.9	174.2 ± 51.0	184.1 ± 46.6	182.8 ± 45.6	179.5 ± 50.7	179.9 ± 48.5	178.5 ± 48.8	173.7 ± 49.2
SBP, mmHg	120.7 ± 13.3	121.4 ± 13.6	122.8 ± 14.8	123.0 ± 16.5	122.0 ± 14.9	121.3 ± 14.0	120.6 ± 13.5	119.8 ± 12.8
DBP, mmHg	78.2 ± 9.9	78.8 ± 10.1	79.9 ± 10.4	79.4 ± 11.3	79.3 ± 10.7	78.8 ± 10.1	78.2 ± 10.0	77.5 ± 9.7
FBS, mg/dL	85.3 ± 26.8	85.6 ± 26.5	89.1 ± 25.4	97.6 ± 40.8	89.6 ± 31.1	87.7 ± 26.4	85.8 ± 24.6	84.4 ± 24.3
**History (yes)**								
Diabetes	12,549 (3.7)	4573 (3.5)	2429 (5.5)	197 (17.2)	640 (9.0)	244 (5.1)	749 (3.9)	331 (2.5)
Hypertension	84,956 (24.9)	35,076 (27.1)	14,070 (32.0)	355 (31.1)	2096 (29.5)	1319 (30.8)	5779 (29.7)	3437 (25.6)
Hyper-lipidemia	96,732 (28.3)	35,183 (27.2)	15,020 (34.1)	362 (31.7)	2222 (31.2)	1471 (34.4)	8070 (32.8)	4475 (28.1)
**Family health history (yes)**								
Cancer	59,358 (17.4)	22,349 (17.3)	9504 (21.6)	215 (18.8)	1491 (21.1)	1017 (21.3)	4005 (20.6)	2527 (18.8)
Stroke	49,622 (14.5)	19,173 (14.8)	8557 (19.4)	198 (17.3)	1233 (17.3)	837 (17.5)	3271 (16.8)	1882 (14.0)

Continuous variables are presented as mean ± standard deviation (SD); categorical values are presented as *n* (%). Percentages may not equal 100 due to rounding. BMI, body mass index; TC, total cholesterol; SBP, systolic blood pressure; DBP, diastolic blood pressure; FBS, fasting blood glucose.

Table S3. Incidence rates of stroke and its subtypes according to smoking cessation and weight change in the study population excluding those with obesity.

	Sustained Smokers (*N* = 341,323)	Non-Smokers (*N* = 129,564)	Long-Term Quitters (*N* = 44,015)	Recent Quitters (*N* = 45,905)				
				Severe Losers (*N* = 1143)	Mild Losers (*N* = 7107)	Maintainers (*N* = 4776)	Mild Gainers (*N* = 19,441)	Severe Gainers (*N* = 13,438)
**Total number of stroke incidences (*N* = 27,755)**								
Number of events	18,008	5100	2416	101	441	266	973	450
Person-years	8,735,982	3,378,502	1,139,563	27,046	178,899	123,292	502,852	345,896
Number of events per 100,000 PY	206	151	212	373	247	216	193	130
**Ischemic stroke (*N* = 21,803)**								
Number of events	14,173	3979	1879	88	346	209	769	360
Person-years	8,763,840	5,852,862	1,143,655	27,179	179,646	123,740	504,637	346,696
Number of events per 100,000 PY	162	68	164	324	193	169	152	104
**Hemorrhagic stroke (*N* = 6564)**								
Number of events	4254	1234	583	13	107	60	220	93
Person-years	8,837,443	3,407,777	1,152,751	27,640	181,559	124,792	508,743	348,813
Number of events per 100,000 PY	48	36	51	47	59	48	43	27
**SAH (*N* = 1957)**								
Number of events	1392	282	138	6	29	11	65	34
Person-years	8,855,264	3,414,320	1,155,878	27,680	182,022	125,125	510,055	349,235
Number of events per 100,000 PY	16	8	12	22	16	9	13	10
**ICH (*N* = 4802)**								
Number of events	3004	979	455	7	83	49	162	63
Person-years	8,847,977	3,409,974	1,153,785	27,745	181,846	124,886	509,199	349,112
Number of events per 100,000 PY	34	29	39	25	46	39	32	18
**Unspecified type of stroke (*N* = 984)**								
Number of events	603	195	113	4	12	7	30	20
Person-years	8,863,128	3,415,427	1,156,369	27,775	182,247	125,166	510,329	349,379
Number of events per 100,000 PY	7	6	10	14	7	6	6	6

PY, person year; SAH, subarachnoid hemorrhage; ICH, Intracerebral hemorrhage.

Table S4. Hazard ratio and 95% confidence interval for stroke and its subtypes according to smoking cessation and weight change in the study population excluding those with obesity.

	Sustained Smokers † (*N* = 341,323)	Non-Smokers (*N* = 129,564)	Long-Term Quitters (*N* = 44,015)	Recent Quitters (*N* = 45,905)				
				Severe Losers (*N* = 1143)	Mild Losers (*N* = 7107)	Maintainers (*N* = 4776)	Mild Gainers (*N* = 19,441)	Severe Gainers (*N* = 13,438)
**Total number of stroke incidences (*N* = 27,755)**								
Model 1 HR (95% CI)	1	0.63 (0.61–0.65)	0.64 (0.61–0.66)	1.22 (1.01–1.49)	0.86 (0.78–0.95)	0.79 (0.70–0.90)	0.80 (0.75–0.85)	0.71 (0.65–0.79)
Model 2 HR (95% CI)	1	0.64 (0.62–0.66)	0.62 (0.60–0.65)	1.11 (0.91–1.35)	0.82 (0.74–0.90)	0.78 (0.69–0.88)	0.80 (0.74–0.85)	0.72 (0.66–0.80)
**Ischemic stroke (*N* = 21,803)**								
Model 1 HR (95% CI)	1	0.62 (0.59–0.64)	0.60 (0.57–0.63)	1.30 (1.06–1.60)	0.83 (0.75–0.92)	0.77 (0.67–0.89)	0.79 (0.73–0.85)	0.73 (0.66–0.82)
Model 2 HR (95% CI)	1	0.62 (0.60–0.64)	0.59 (0.56–0.61)	1.18 (0.96–1.45)	0.78 (0.70–0.87)	0.75 (0.66–0.87)	0.79 (0.73–0.84)	0.74 (0.67–0.82)
**Hemorrhagic stroke (*N* = 6564)**								
Model 1 HR (95% CI)	1	0.69 (0.65–0.74)	0.75 (0.69–0.82)	0.74 (0.43–1.27)	0.97 (0.80–1.18)	0.82 (0.64–1.10)	0.80 (0.70–0.92)	0.61 (0.50–0.75)
Model 2 HR (95% CI)	1	0.71 (0.66–0.76)	0.74 (0.68–0.81)	0.70 (0.40–1.20)	0.94 (0.78–1.14)	0.81 (0.63–1.04)	0.80 (0.70–0.92)	0.62 (0.50–0.76)
**SAH (*N* = 1957)**								
Model 1 HR (95% CI)	1	0.51 (0.45–0.58)	0.63 (0.53–0.75)	1.20 (0.62–1.30)	0.90 (0.62–1.30)	0.50 (0.28–0.91)	0.76 (0.60–0.98)	0.65 (0.46–0.91)
Model 2 HR (95% CI)	1	0.51 (0.44–0.57)	0.63 (0.53–0.75)	1.22 (0.55–2.72)	0.90 (0.62–1.30)	0.50 (0.28–0.91)	0.77 (0.60–0.98)	0.65 (0.47–0.92)
**ICH (*N* = 4802)**								
Model 1 HR (95% CI)	1	0.76 (0.71–0.82)	0.78 (0.71–0.86)	0.53 (0.25–1.12)	1.02 (0.82–1.27)	0.92 (0.69–1.22)	0.82 (0.70–0.96)	0.59 (0.46–0.76)
Model 2 HR (95% CI)	1	0.79 (0.73–0.85)	0.77 (0.69–0.85)	0.49 (0.23–1.03)	0.98 (0.78–1.21)	0.90 (0.68–1.20)	0.82 (0.70–0.96)	0.60 (0.47–0.72)
**Unspecified type of stroke (*N* = 984)**								
Model 1 HR (95% CI)	1	0.72 (0.61–0.84)	0.86 (0.70–1.05)	1.39 (0.52–3.72)	0.68 (0.39–1.21)	0.61 (0.29–1.29)	0.73 (0.50–1.04)	0.97 (0.62–1.51)
Model 2 HR (95% CI)	1	0.72 (0.61–0.85)	0.84 (0.69–1.03)	1.25 (0.47–3.5)	0.65 (0.37–1.49)	0.60 (0.8–1.26)	0.73 (0.50–1.05)	1.00 (0.62–1.52)

SAH, subarachnoid hemorrhage; ICH, Intracerebral hemorrhage. Model 1: adjusted for age, body mass index. Model 2: further adjusted for exercise, alcoholic drinks, medical history (diabetes, hyperlipidemia, hypertension), and family medical history (cancer, stroke). † This group served as the reference group.
